# Supplementary material for: Caudatin Isolated from Cynanchum auriculatum Inhibits Breast Cancer Stem Cell Formation via a GR/YAP Signaling
Source: Biomolecules. 2020 Jun 18;10(6):925. doi: 10.3390/biom10060925 (PMC7355644; doi:10.3390/biom10060925)
Supplement: Supplementary file 1 [file biomolecules-10-00925-s001.zip › biomolecules-811005-supplementary.docx]

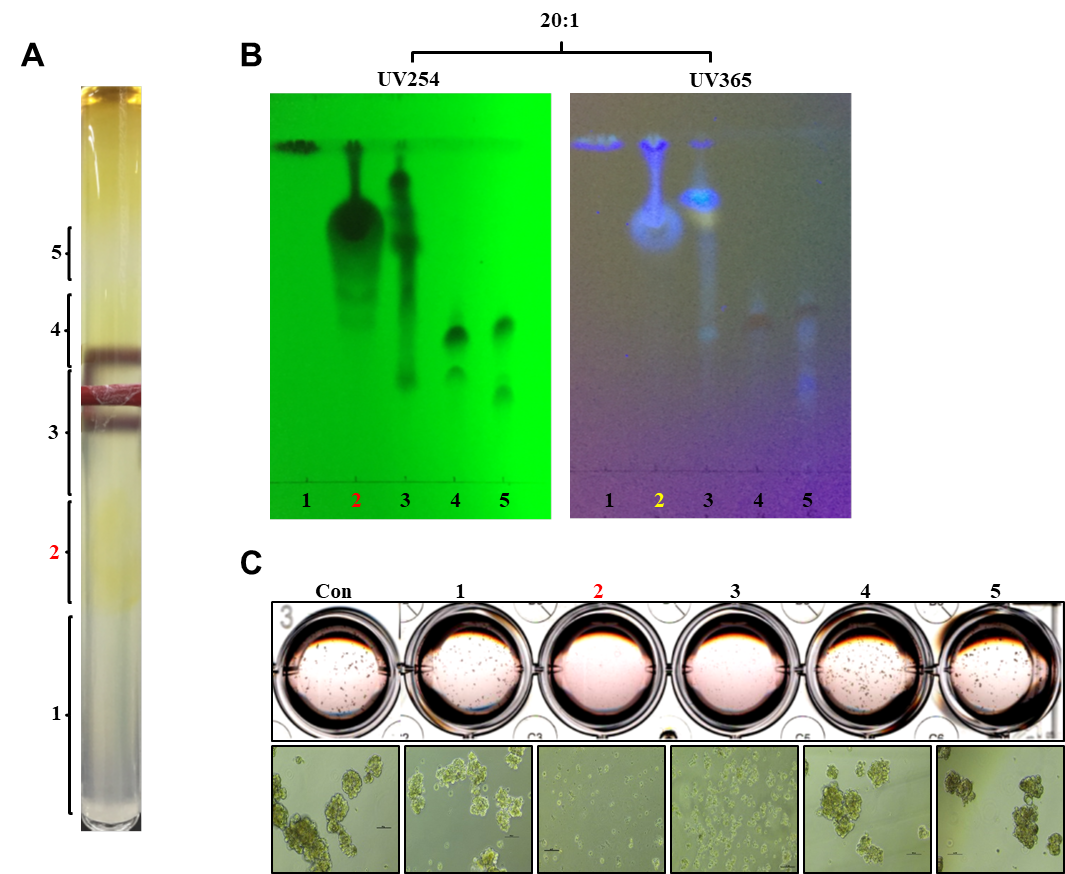


**Figure S1.** The purification procedure of cancer stem cell inhibitor derived from *C. auriculatum* using SiO_2_ gel chromatography. (**A**) The sample was isolated by silica gel chromatography with a solvent mixture [CHCl_3_ : MeOH (20:1)]. (**B**) TLC plate analysis of the purified sample (CHCl_3_ : MeOH = 20:1). Active fraction: #2. (**C**) Mammosphere formation assay using the purified fraction #2.

.

**
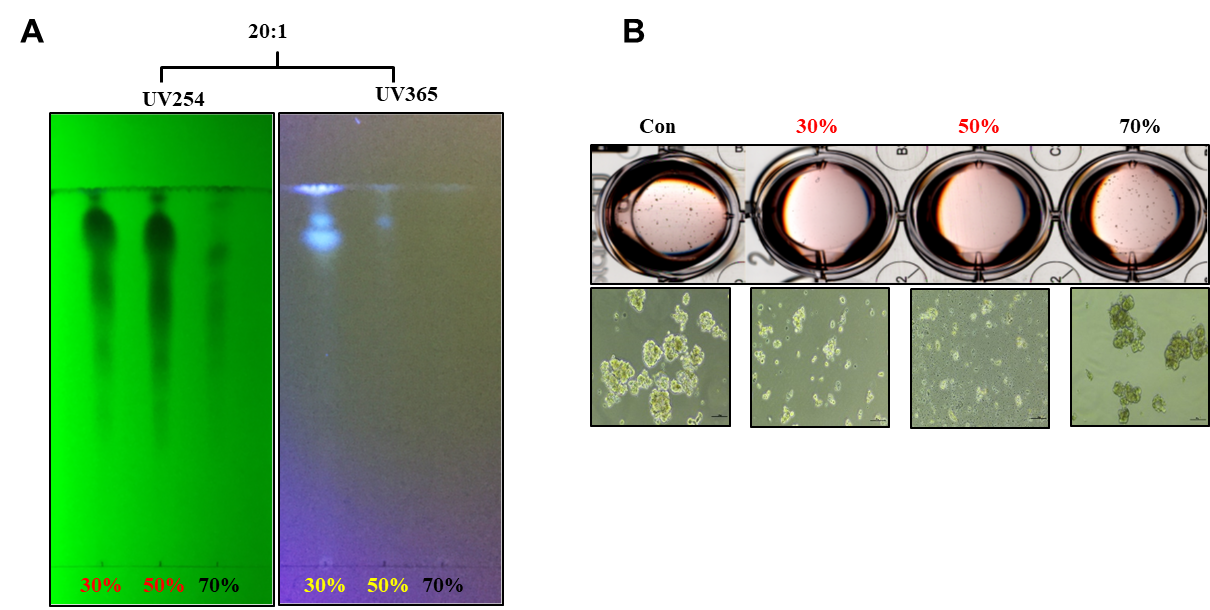
**

**Figure S2.** Purification procedure of cancer stem cell inhibitor in *C.auriculatum* using Reversed-Phase C_18_ (ODS) gel chromatography. (**A**) Thin-layer chromatography analysis of the partial-purified sample from ODS resin. Purified-sample were spotted and developed in chloroform: methanol *(*20:1). UV was used to detect the samples. (**B**) Mammosphere formation assay using breast cancer stem cell derived from MDA-MB-231 cells using partial-purified sample.


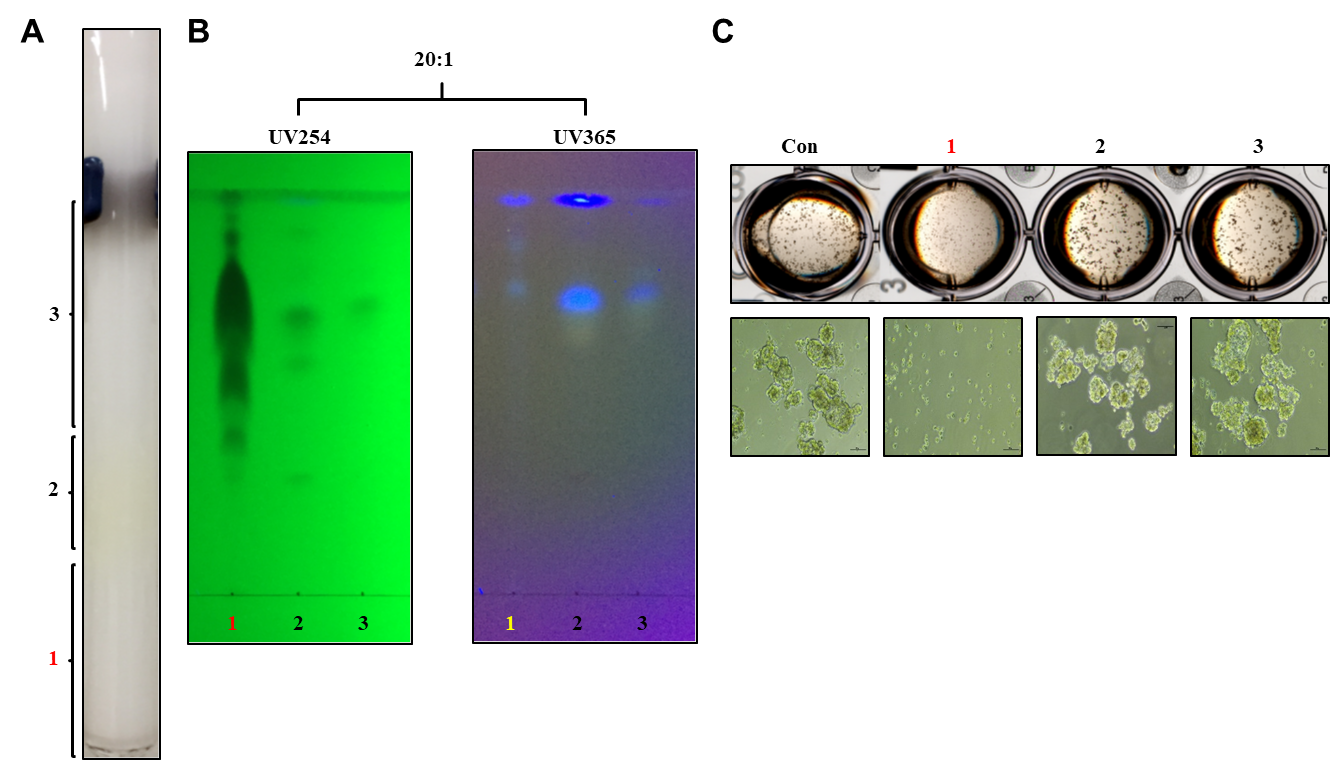


Figure S3. Purification procedure of cancer stem cell inhibitor in *C. auriculatum* using Sephadex LLH-20 gel chromatography. (**A**) The sample was isolated by Sephadex LH-20 chromatography with MeOH. (**B**) TLC plate analysis of the purified sample (CHCl_3_ : MeOH = 20:1). Active fraction: #1. (**C**) Mammosphere formation assay using the purified fraction #1.


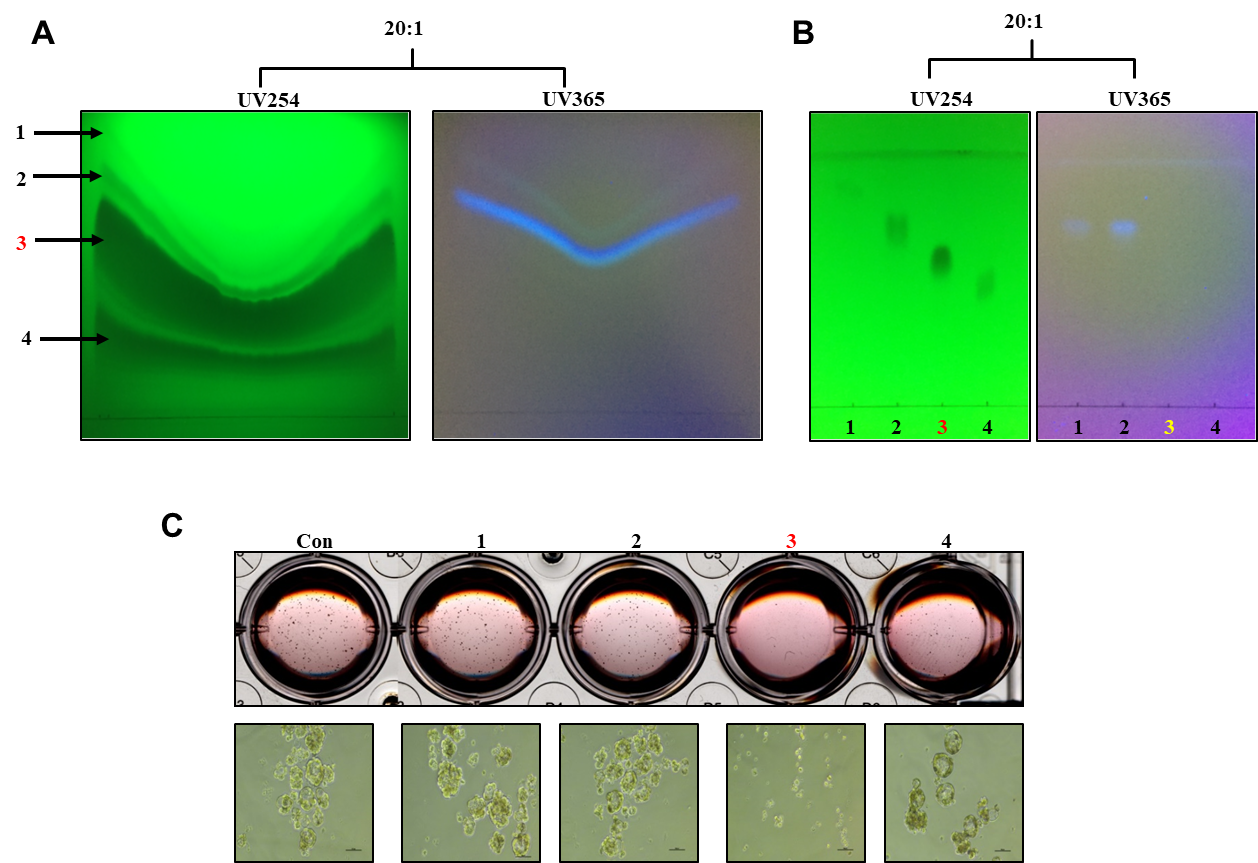


Figure S4. Purification procedure of cancer stem cell inhibitor in *C. auriculatum* using a preparative thin layer chromatography. (**A**) Preparatory TLC chromatography containing fractions 1. (**B**) TLC plate analysis of the prepared TLC bands after the samples were scraped and purified (CHCl_3_ : MeOH = 20:1). Active fraction: #3. (**C**) Mammosphere formation assay using the purified fraction #3.


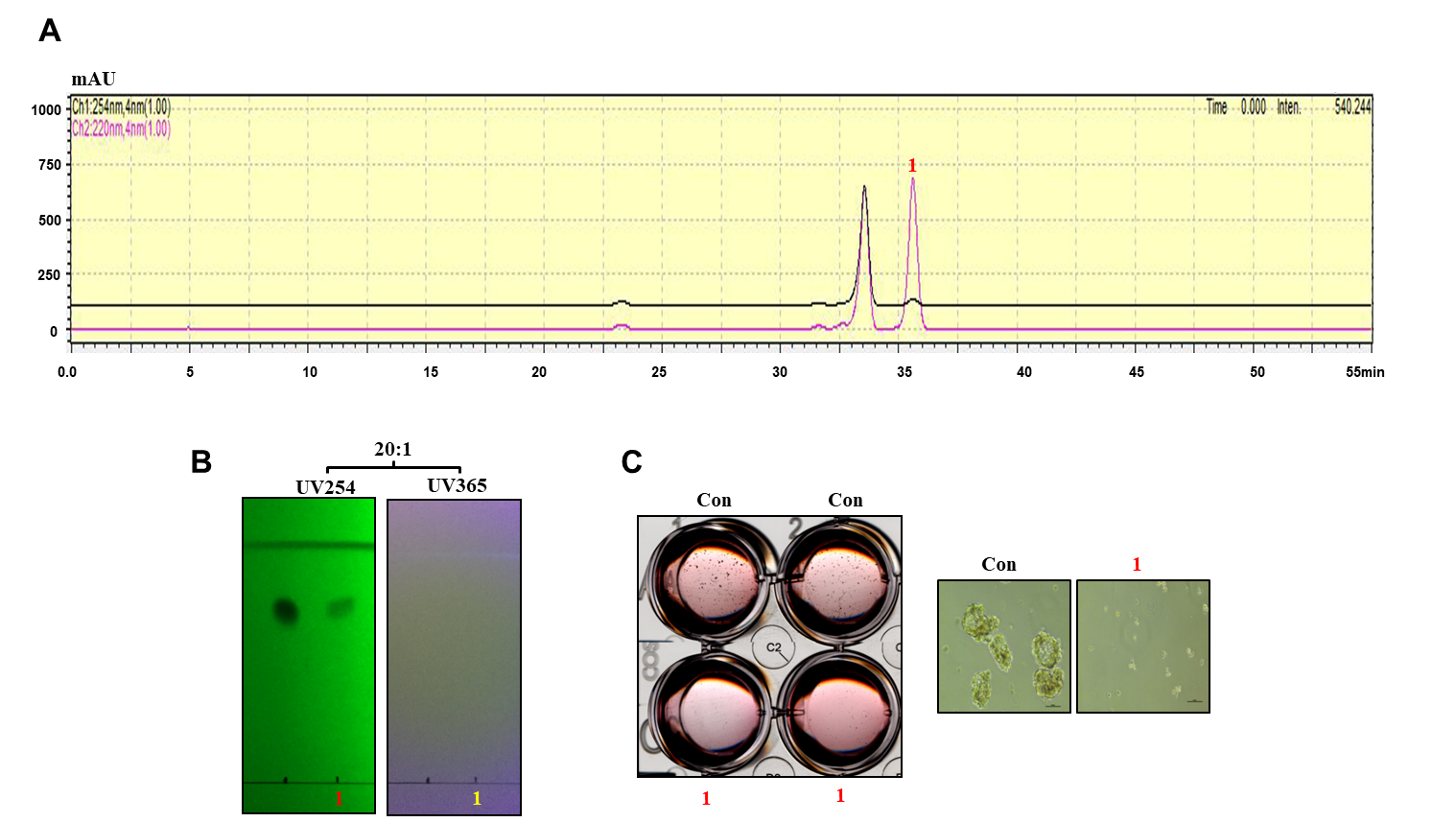


Figure S5. Major fractions collected using High Performance Liquid Chromatography in two wavelength. Samples were collected based on 254 and 220nm wavelength. (**A**) Assessment of the major fractions using HPLC at two wavelengths. Samples were collected based on the 254 and 220 nm wavelengths. (**B**) TLC plate analysis of the purified sample (CHCl_3_ : MeOH = 20:1). (C) Mammosphere formation assay using the purified sample (fraction #1).


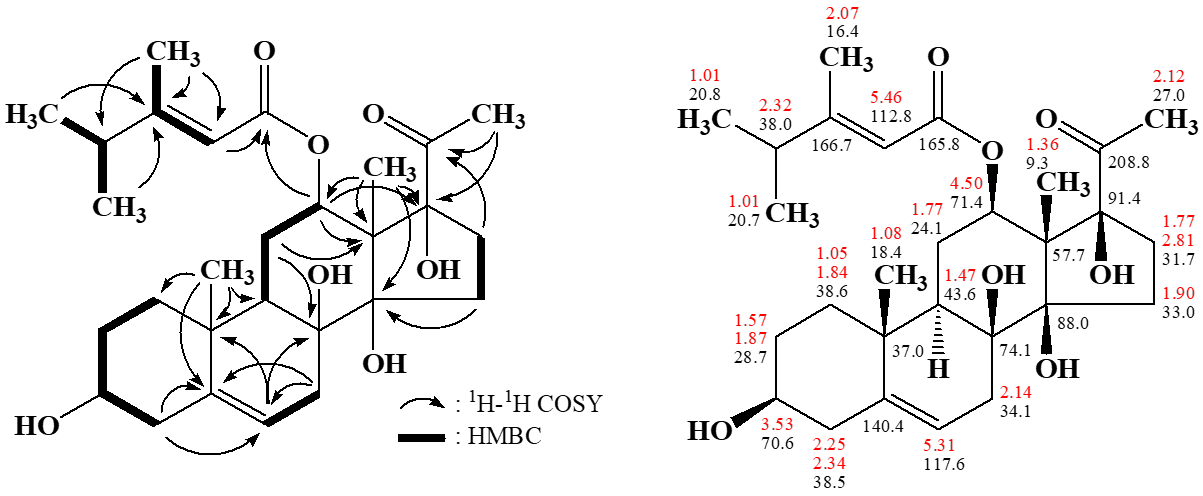


**Figure S6.** Two-dimensional NMR dada and ^1^H (red) and ^13^C (black) NMR peak assignments of caudatin (C_28_H_42_O_7_; 490).


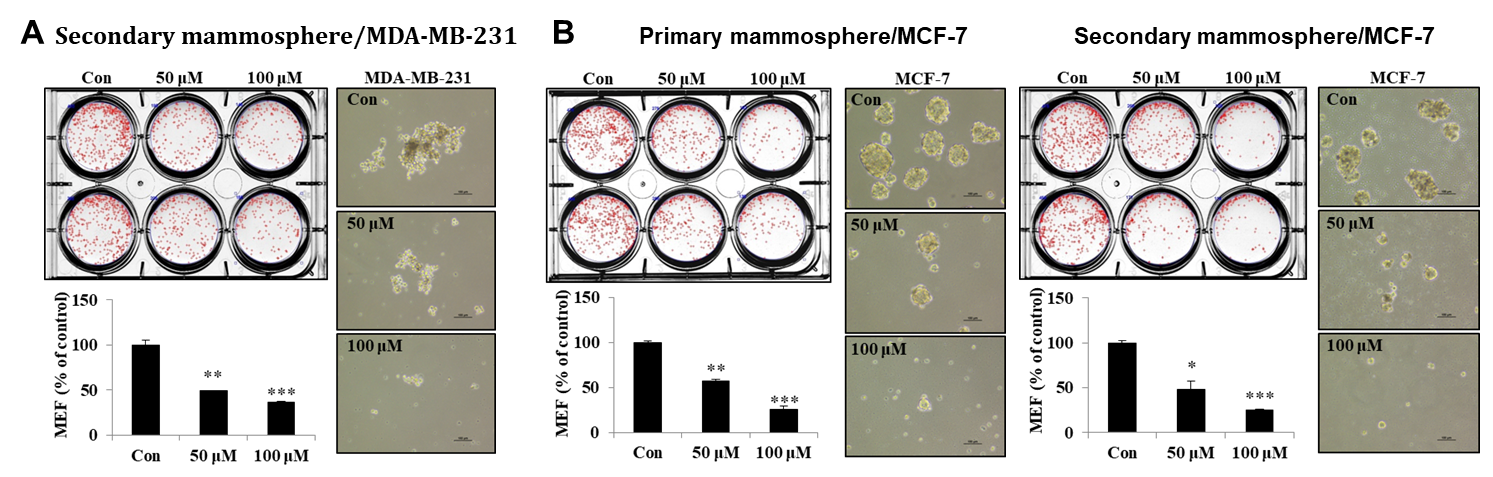


**Figure S7.** The inhibitory effect of caudatin on mammosphere formation is also observed in secondary (MCF-7 cells and MDA-MB-231 cell) and primary mammosphere assay (MCF-7 cells). **A**; 2^nd^ mammosphere derived from MDA-MB-231 cells, **B**; 1st and 2nd mammosphere derived from MCF-7.
